# Hydroalcoholic extract of *Buxus sempervirens* shows antiproliferative effect on melanoma, colorectal carcinoma and prostate cancer cells by affecting the autophagic flow

**DOI:** 10.3389/fphar.2023.1073338

**Published:** 2023-02-20

**Authors:** Anna Rita Volpe, Marco Carmignani, Patrizia Cesare

**Affiliations:** Section of Pharmacology and Toxicology, Department of Life, Health and Environmental Sciences, University of L'Aquila, L'Aquila, Italy

**Keywords:** *Buxus sempervirens*, autophagy, melanoma, colorectal carcinoma, prostate cancer

## Abstract

*Buxus sempervirens* (European Box, Buxaceae, boxwood) has been used in folk medicine to treat rheumatism, arthritis, fever, malaria and skin ulceration while, in recent years, interest has grown on possible employment of boxwood extracts in cancer therapy. We studied the effect of hydroalcoholic extract from dried leaves of *Buxus sempervirens* (BSHE) on four human cell lines (BMel melanoma cells, HCT116 colorectal carcinoma cells, PC3 prostate cancer cells, and HS27 skin fibroblasts) to ascertain its possible antineoplastic activity. This extract inhibited proliferation of all cell lines in different degree as shown, after 48 h-exposure and MTS assay, by the values of GR_50_ (normalized growth rate inhibition_50_) that were 72, 48, 38, and 32 μg/mL for HS27, HCT116, PC3 and BMel cells, respectively. At the above GR_50_ concentrations, 99% of all studied cells remained vital showing accumulation of acidic vesicles in the cytoplasm, mainly around nuclei, whereas a higher extract concentration (125 μg/mL) was cytotoxic causing, after 48 h-exposure, death of all BMel and HCT116 cells. Immunofluorescence showed microtubule-associated light chain three protein (LC3, a marker for autophagy) to be localized on the above acidic vesicles when cells were treated for 48 h with BSHE (GR_50_ concentrations). Western blot analysis revealed, in all treated cells, a significant increase (2.2–3.3 times at 24 h) of LC3II, i.e., the phosphatidylethanolamine conjugate of the cytoplasmic form LC3I that is recruited in autophagosome membranes during autophagy. Such increase was accompanied, in all cell lines treated for 24 h or 48 h with BSHE, by a significant increment (2.5–3.4 times at 24 h) of p62, an autophagic cargo protein undergoing degradation during the autophagic process. Therefore, BSHE appeared to promote autophagic flow with its following blockade and consequent accumulation of autophagosome or autolysosomes. The antiproliferative effects of BSHE also involved cell cycle regulators such as p21 (HS27, BMel and HCT116 cells) and cyclin B1 (HCT116, BMel and PC3 cells) whereas, among apoptosis markers, BSHE only decreased (30%–40% at 48 h) the expression of the antiapoptotic protein survivin. It was concluded that BSHE impairs autophagic flow with arrest of proliferation and death in both fibroblasts and cancer cells, being the latter much more sensitive to these effects.

## 1 Introduction

Medical plants have been used for millennia to treat a wide spectrum of diseases and, in recent history, plant-derived natural products continue to represent either useful therapeutic agents in many pathological conditions or springboard for development of new chemical entities. Natural drugs are often provided with high specificity and selectivity of action and, in particular, many of them interfere with the cell cycle processes, thus constituting an efficacious antineoplastic approach (Cragg et al., 2009).

*Buxus sempervirens L* (European Box, Buxaceae, boxwood) is an evergreen ornamental plant very widespread in Europe and decoctions of its leaves have been used in folk medicine to treat rheumatism, arthritis, fever, malaria and skin ulceration (Leporatti et al., 1985; Neves et al., 2009). Steroid compounds have been identified in extracts from this plant (Rahman et al., 1992; Loru et al., 2000), while the alkaloid fraction of extracts from *B. sempervirens L* (BS) leaves showed a selective *in vitro* activity against *Plasmodium falciparum* (Althaus et al., 2014). Recently, Szabó et al. (2021) characterized 25 alkaloids from leaves of BS, being five of them active against *P. falciparum* and/or *Trypanosoma brucei rhodesiense*. Moreover, alkaloids from BS (Orhan et al., 2011) and *Buxus natalensis* (Matochko et al., 2010) evidenced anticholinesterase activity, and alkaloids from *Buxus hyrcana* revealed potent immunosuppressive properties as shown by their inhibitory effects on oxidative burst, chemotaxis, T-cell proliferation and cytokine production (Mesaik et al., 2010). In this regard, buxusemine A and buxanoldine, two alkaloids present in the methanolic extract of BS, were protective when assayed in a model of doxorubicin-induced cardiac injury (Xiang et al., 2021). Moreover, aglycone and glucoside flavonoid-enriched extracts from leaves of BS exhibited antidiabetic effect in streptozotocin-treated rats (Ajebli and Eddouks, 2020).

Only few studies dealt with the possible anticancer activity of BS extracts. One of the first works on this subject reported that acetonic extract of BS was provided *in vitro* with antitumor activity against breast cancer through induction of autophagic cell death and apoptosis (Ait-Mohamed et al., 2011). More recent papers focused on cyclovirobuxine D, a steroidal alkaloid present in various species of boxwood including BS for which antitumor activity was reported against several neoplasms including breast, lung and colorectal cancers, hepatocellular carcinoma and glioblastoma multiforme (Jiang et al., 2020; Wang et al., 2020; Zhang et al., 2020; Zhou et al., 2020; Zeng et al., 2021). Such steroidal alkaloid appeared to act on different signalling pathways also regulating apoptosis and cell proliferation and migration.

The present study was addressed to evaluate the effect of hydroalcoholic extract of BS (BSHE) on three human tumoral cell lines (BMel melanoma cells, HCT116 colorectal carcinoma cells and PC3 prostate cancer cells) in comparison with its effect on normal human cells (HS27 skin fibroblasts).

## 2 Materials and methods

### 2.1 Cell cultures

The Hs27 normal human dermal fibroblast cell line (ATCC 1634-CRL), the HCT116 human colorectal carcinoma cell line (ATCC CCL-247) and the PC3 human prostate cancer cell line (ATCC 11435-CRL) were purchased by American Type Culture Collection (Manassas, Va), while the BMel human melanoma cell line was a gift from Prof. P.G.Natali (“Regina Elena” Institute for Cancer Research, Rome, Italy). All cell types were grown in Dulbecco’s modified Eagle medium, supplemented with 10% fetal bovine serum, 2 mM L-glutamine, 100 IU/mL penicillin and 100 μg/mL streptomycin, and maintained at 37°C in humidified atmosphere with 5% CO_2_. Medium was replaced every 3 days and cells were detached and sub-cultured when approximately 90% confluence was reached. All cell culture materials were purchased by Euroclone (Milan, Italy).

### 2.2 Boxwood hydroalcoholic extract preparation

Plant extract was provided by “Laboratori Erboristici Caira” (Villa Latina, Italy). Briefly, leaves of *B. sempervirens* were collected around Villa Latina in September. Fresh leaves were dried and dry leaves (100 g) were macerated in 200 mL of hydroalcoholic solution (50% ethanol) at room temperature and then clarified by filtration. The hydroalcoholic plant extract (50 mg dry weight/mL), not subjected to concentration procedures, was stored at room temperature in the dark and diluted in culture medium when used. The expert who identified the plant was Dr. Marco Leonardi, university researcher of Systematic Botany at the Environmental Sciences Section, Dept. of Life, Health and Environmental Sciences, University of L’Aquila, Italy, being the voucher specimen of the plant specimen deposited in his personal herbarium (Pharmacology Lab) with the acronym PC 001-20.

### 2.3 Cell proliferation and viability

Trypan blue dye exclusion test (TBDET) and MTS assay [3-(4,5-dimethylthiazol-2-yl)-5-(3-carboxymethoxyphenyl)-2-(4-sulfophenyl)-2H-tetrazolium; CellTiter 96 Aqueous One Solution reagent, Promega, Madison, WI] were employed in determining viability of the studied cells whose proliferation was assessed following exposure for various times to different concentrations of BSHE. Two vitality tests were adopted since they may have some single limitations due to the manual execution (TBDET) and to the interference with substances present in the system and to an increased number of mitochondria leading to overestimate the number of cells (MTS) (Kok et al., 2007; Wang et al., 2010; McGowan et al., 2011).

For TBDET, cells (in 1.5 mL culture medium/well) were seeded into 6-well plates at approximately 10,000 cells/cm^2^ for Hs27 and PC3 lines, and 5,000 cells/cm^2^ for HCT116 and BMel lines. After 24 h, cells were exposed to scalar concentrations of BSHE, diluted in culture medium, ranging from 3.9 to 500 μg/mL. Cell counting was performed every 24 h by using a Neubauer hemocytometer and a Nikon Eclipse TS 100 inverted microscope equipped with a phase contrast objective. Cell viability was assessed by removing cells from the plates with 0.05% trypsin-0.02% EDTA solution and by combining 20 µL aliquots of this cell suspension with 20 µL of 4% trypan blue dye solution. The dye stained the injured cells but not the uninjured ones, while a diffuse cytoplasmic staining indicated cell death (Tolnai, 1975). Three wells for each BSHE concentration were counted and this procedure was repeated three times. Two controls were predisposed, the first consisting of cells not exposed to BSHE, the second consisting of cells exposed to vehicle alone (i.e., aliquots of hydroalcoholic solutions with a maximum ethanol concentration of 0.5%). No difference in cell proliferation and vitality resulted between the two controls so that only data from the first one were reported.

For MTS assay, cells (in 100 µL culture medium/well) were seeded in 96-well plates (1,500 cells/cm^2^ for HS27 and PC3 lines, 1,000 cell//cm^2^ for BMel and HCT 116 lines), maintained 24 h for attachment and then exposed to suitable concentrations of BSHE. Culture medium was replaced after 24, 48, and 72 h, and 20 µL of MTS reagent were added. After 60 or 120 min, absorbance was determined at 490 nm by a Microplate reader model 680 (Biorad). A blank experiment, detecting background absorbance of cell-free wells, was performed in parallel and the resulting values were subtracted from those detected in cell-containing wells to obtain net absorbance. Each condition was reproduced in quadruplicate and the experiment was repeated three times. The controls were as for TBDET.

For quantification of drug response, the number of viable cells (or surrogates such as net A_490nm_ using MTS assay) in the presence of BSHE divided by cell counts for untreated controls was fitted to a sigmoidal curve to compute the concentration of drug at which the cell count is half of the control (IC_50_). However, this traditional parameter (such as AUC and E_max_) is confounded by the different numbers of cell divisions taking place during an assay because of natural differences in proliferation rate, variations in growth conditions or changes in the duration of an experiment, thus varying dramatically independently from any changes in the underlying biology. Therefore, we used a recently proposed new method for parameterizing drug response, i.e., the normalized growth rate inhibition (GR), which is based on comparing growth rates in the presence and absence of drug. In this regard, GR at time *t* in the presence of drug at concentration *c* will be:

$$GR\left(c\right)=2^{k\left(c\right)/k\left(0\right)}-1$$

where *k(c)* is the growth rate of drug-treated cells and *k(0)* is the growth rate of untreated (or vehicle-treated) control cells. In practice, growth rate can be evaluated by considering both number of cells at the beginning of the treatment as just prior to drug exposure (*x*_*0*_) and number of cells at the end of the assay in an untreated (or vehicle-treated) control well [x (0)] and in a drug-treated well [x(c)] (Hafner et al., 2016; 2017; Clark et al., 2017). The GR value is thus:

$$GR\left(c\right)=2^{\frac{\log_{2}\left(x\left(c\right)/x_{0}\right)}{\log_{2}\left(x\left(0\right)/x_{0}\right)}}-1$$

Therefore, the GR value represents the ratio between growth rates under treated and untreated conditions normalized to a single cell division. Such value relates directly to response phenotype indicating partial growth inhibition (when it lies between 0 and 1), complete cytostasis (when it equals 0) or cell death (when it lies between 0 and −1). Parameterization of GR dose-response curves yields, *inter alia*, GR_50_, analogous to EC_50_, representing the concentration at which GR(c) = 0.5 and the pertubagen has half of its maximal effect on cell growth, while being largely independent of cell division rate and assay duration. On the other hand, GR metrics appear to be more robust than traditional metrics in assessing the effects of drugs on cell signalling and growth. In the present study, cell number was determined in TBDET by direct counting or, in MTS assay, by using the net absorbance at 490 nm as surrogate for direct cell counting. Online *GRcalculator* tools ( *http://www.grcalculator.org* ) were employed for calculation, analysis and visualization of dose-response data using GR approach and for comparison of GR and traditional metrics (Clark et al., 2017).

### 2.4 Western immunoblot analysis

Cells were seeded in T75 culture flasks at a density of l × 10^4^ cells/cm^2^, maintained 24 h for attachment and then treated or not with BSHE, whose GR_50_ concentrations were referred to the 48 h-exposure).

After 24 and 48 h of incubation, control and treated cells were harvested and lysed in RIPA buffer [containing 1 mM PMSF (phenyl-methyl-sulfonyl fluoride) and (1 μg/mL each) aprotinin, pepstatin A and chymostatin (SIGMA-Aldrich)] by passing cell suspensions through a syringe equipped with 15 G, 25 G and 30 G needles (10 times each). The soluble protein fraction was then collected after centrifugation at 13,000 *g* for 30 min at 4°C and total protein content was assessed by Pierce BCA Protein Assay Kit (Thermo Scientific). Samples were then diluted (1:1 v/v) in the sample buffer (125 mM Tris-HCl, 20% glycerol, 10% β-mercaptoethanol, 4% sodium dodecyl sulphate, bromophenol blue, pH 6.8); denatured samples (15 µg total proteins) were run through 12% polyacrylamide denaturing gels and proteins were transferred onto methanol-activated polyvinylidene fluoride (PVDF) sheets (Millipore) by wet electrophoretic transfer (Laemmli, 1970; Towbin et al., 1979). Non-specific binding sites were blocked with 5% non-fat dried milk in TBS-T (10 mM Tris-HCI, 150 mM NaCl and 0.05% Tween-20, pH 7.5) for 60 min. Afterwards, samples were incubated overnight at 4°C with different primary antibodies (dilution l:1,000 each) in 5% non-fat dried milk in TBS-T. Primary antibodies were a) from Cell Signaling Technology, Inc, Danvers, MA, United States, i.e., anti-p62/SQSTM1Ab (cat. 88588), anti-p21 Waf1/Cip1 Ab (cat. BK 2947T), anti-survivin Ab (cat. BK 2808T), anti-p27 Kip1 Ab (cat. BK3698T) and anti-beclin-1 Ab (cat. BK 3495T) b) from Immunological Sciences, Rome, Italy, i.e., anti-cyclin B1 Ab (cat. AB84359), anti-p53 Ab (cat. AB83528), anti-Bcl_2_ Ab (cat. AB 82346), anti-BAX Ab (cat. 82329) and anti-cyclin D1 Ab (cat. AB82346) c) from Thermo Fisher Scientific Waltham, MA, United States, i.e., anti-cyclin E Ab (cat. MA5-14336) d) from Novus biologicals Littleton, CO, United States, i.e., anti-LC3B Ab (cat. NB100-2220) e) from Santa Cruz Biotechnology, Inc, Santa Cruz, CA, United States, i.e., anti-β-actin Ab (cat. sc47778).

Finally, PVDF sheets were washed three times with TBS-T (10 min each), incubated with appropriate horseradish peroxidase-conjugated secondary antibody for 2 h at room temperature (dilution 1:1,000), and rinsed 3 times (5 min each) with TBS-T and once with TBS (i.e., TBS-T without 0.05% Tween-20).

Images of the specific immune complexes were revealed, acquired and analyzed by using Enhanced Chemi-luminescent Substrate Kit (Euroclone, Milan, Italy), Alliance Q9 hardware (UVItec Limited, Cambridge, United Kingdom), and Total Lab TL120 software (TotalLab, Newcastle upon Tyne, United Kingdom). Densitometry values were normalized for β-actin and experiments were performed in triplicate. Results were given as protein expression relative to control (relative intensity).

### 2.5 Acridine orange (AO) staining

Cells were seeded in coverslips dishes at a density of l × 10^4^ cells/cm^2^, maintained 24 h for attachment and then treated or not with BSHE (whose GR50 concentrations were those referred to the 48 h-exposure). After 48 h-incubation, cells were washed twice with PBS, stained with 4 μg/mL AO for 10 min and washed again with PBS. The coverslips were overturned on microscope slides and immediately observed under fluorescence microscopy (Axio Imager A2 Zeiss). Images were acquired using a color camera (DFC320 Leica). AO is a metachromatic dye whose luminescence wavelength is strongly dependent on its concentration. In live cells, AO is accumulated by acidic vesicles yielding strong orange or red signals; it is also provided with affinity for nucleic acids yielding less intense green fluorescence (Pierzynska-Mach et al., 2014). The vesicle red fluorescence intensity was quantified by using Fiji-ImageJ image processing software (Schindelin et al., 2012). At least 50 cells per each experimental condition were analysed, while corrected total cell fluorescence (CTCF), of both BSHE-untreated and BSHE-treated cells, was obtained as follows: CTCF = Integrated Density - (Area of selected cell x Mean fluorescence of background readings). (https://theolb.readthedocs.io/en/latest/imaging/measuring-cell-fluorescence-using-imagej.html#measuring-cell-fluorescence-using-imagej).

### 2.6 Immunofluorescence

Cells were seeded in coverslips dishes at a density of 1 × 10^4^ cells/cm^2^, maintained 24 h for attachment and then treated with BSHE extract (whose GR_50_ concentrations were those referred to the 48 h-exposure).

After 48 h-incubation, cells were washed twice with PBS, fixed with 4% paraformaldehyde and permeabilized with PBS containing 0.5% Triton X-100. Non-specific binding sites were blocked following a 30 min-exposure to 3% bovine serum albumin (BSA) in PBS-T (PBS and 0.05% Tween-20). Afterwards, cells were incubated with rabbit anti-LC3B antibody (dilution 1:200 in PBS and 1% BSA) at 4°C overnight. Cells were then washed three time with PBS-T and incubated with secondary Dy Light 488-conjugated anti-rabbit antibodies (dilution 1:1,000 in PBS and 1% BSA) in the dark at 4°C for 1 h and washed again with PBS-T.

Coverslips (overturned on microscope slides) were mounted with Pro Long Diamond Antifade containing DAPI (4′,6-Diamidino-2-Phenylindole, Dihydrochloride) (Thermo Fisher Scientific) and observed under fluorescence microscopy (Axio Imager A2 Zeiss). Images were acquired using a black and white camera (Leica, DFC350FX).

### 2.7 Statistics

Kolmogorov-Smirnov test of goodness of fit (*p*-value >0.05) indicated no significant departure from normal distribution for the obtained data. Therefore, parametric tests were used for detecting differences at the 0.05 level of significance. Data were expressed as mean ± S.E.M. or S.D. of *n* independent experiments. Differences between mean values were estimated by the Student’s t-test. Post-hoc comparisons were performed using the Dunnett test of GraphPad Prism ver. 6.1 software (United States).

## 3 Results

### 3.1 Effect of BSHE on cell proliferation and viability

**FIGURE 1:**
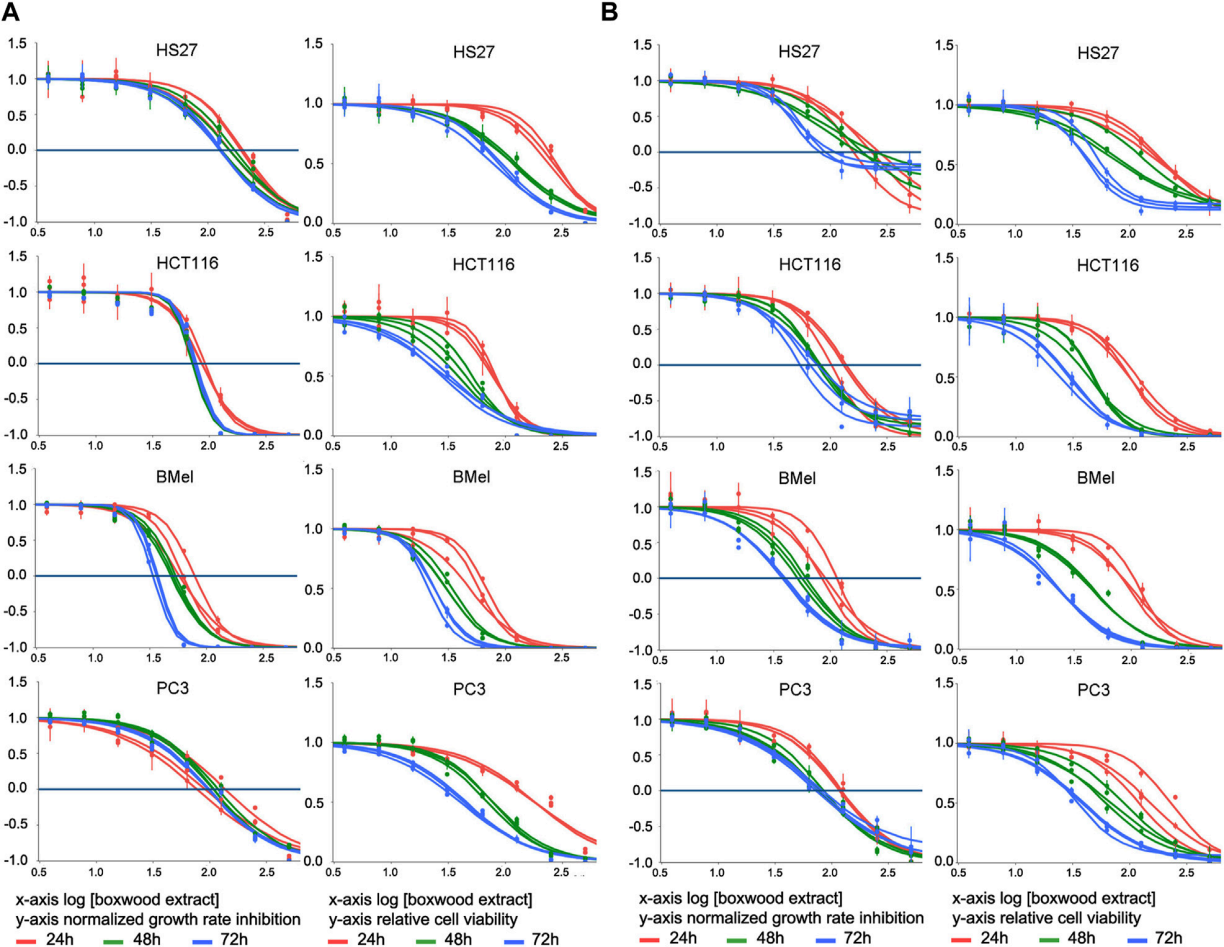
Effect of treatment for 24–48–72 h with *Buxus sempervirens* hydroalcoholic extract (BSHE) on cell proliferation and viability as determined by the trypan blue exclusion test **(A)** and the MTS assay **(B)**. HS27: normal human dermal fibroblasts; HCT116: human colorectal carcinoma cells; BMel: human melanoma cells; PC3: human prostate cancer cells. Graphs on the left columns of both panels show the effect of various BSHE concentrations (*x*-axis, logarithmic values) on normalized growth rate inhibition (GR, see Materials and Methods) as reported on *y*-axis. Graphs on the right columns of both panels show the effect of various BSHE concentrations (*x*-axis, logarithmic values) on relative cell viability (*y*-axis), i.e., number of viable cells (or net A490 nm using MTS assay) under BSHE treatment *versus* BSHE-untreated controls. BSHE concentrations were 3.9, 7.8, 15.6, 31.2, 62.5, 125, 250, and 500 μg/mL. Means ± S.D. were reported (n = 3 for the trypan blue exclusion test, n = 4 for the MTS assay). Graphs were obtained from the online tool GR calculator (www.grcalculator.org; see Materials and Methods).

We first examined the effect of BSHE on cell proliferation under assessment of cell viability by either TBDET or MTS assay. As reported in Materials and Methods, the antiproliferative effect of BSHE in the studied cell lines was evaluated not only by IC50 (concentration at which the cell number is half the control) but also by a novel approach consisting in the evaluation of GR, i.e., normalized growth rate inhibition that takes into account cell growth rate in the presence and absence of drug treatment. GR allows to overcome the interference that the different growth speeds of different cell lines exert on traditional metrics (like IC_50_, E_max_ and AUC) not taking into account the number of recurring cell divisions. Parameterization of GR data through the online tool GR calculator (see above) yields, *inter alia*, GR_50_, GR_max_ and h_GR_ (Hill Slope) values (minimally dependent on cell division rate and assay duration) along with traditional metric parameters (such as relative cell vitality and IC_50_) and concentration-response curves from which GR_50_ and IC_50_ were obtained (Figures 1A, B; Table 1). In this regard, such curves obtained with GR metrics or traditional metrics had a high coefficient of determination (*R* ^2^ > 0.98).

**TABLE 1:** GR50 and IC50 values (µg/mL) of *Buxus sempervirens* hydroalcoholic extract in HS27 normal human dermal fibroblasts, HCT116 human colorectal carcinoma cells, BMel human melanoma cells and PC3 human prostate cancer cells at different exposure times.

Cell line	Assay	24 h		48 h		72 h	
		GR_50_	IC_50_	GR_50_	IC_50_	GR_50_	IC_50_
HS27	**Trypan Blue**	106 ± 20	260 ± 20	81 ± 13	119 ± 5	71 ± 4	86 ± 7
	**MTS**	107 ± 11	202 ± 27	**72** ± 15	111 ± 36	46 ± 3	50 ± 7
HCT116	**Trypan Blue**	62 ± 5*	80 ± 3***	58 ± 1*	45 ± 7***	61 ± 1*	30 ± 2***
	**MTS**	76 ± 8*	104 ± 10***	**48** ± 2*	46 ± 3*	37 ± 3*	28 ± 3**
BMel	**Trypan Blue**	45 ± 10**	58 ± 10***	35 ± 2**	32 ± 2***	28 ± 1***	23 ± 2***
	**MTS**	61 ± 15*	106 ± 38***	**32** ± 8*	46 ± 1*	20 ± 1***	23 ± 1**
PC3	**Trypan Blue**	42 ± 15*	180 ± 1**	57 ± 3*	75 ± 5***	46 ± 2***	43 ± 3***
	**MTS**	65 ± 3**	168 ± 55	**38** ± 5*	73 ± 14	32 ± 2**	38 ± 2*

Cell proliferation and viability were determined by the trypan blue exclusion test and the MTS assay. GR50 and IC50 values were obtained by online tool GR calculator (www.grcalculator.org); GR50 is the drug concentration at which the normalized growth rate inhibition (GR) = 0.5; IC50 is the drug concentration at which the cell number is half of the control; see Materials and Methods. Means ± S.D are reported (n = 3 for the trypan blue exclusion test, n = 4 for the MTS assay). Data were analyzed by the Student’s t-test comparing the values of GR50 and IC50 for cancer cells *versus* those for HS27 normal cell line. *p < 0.05; **p < 0.01; ***p < 0.001.

BSHE inhibited proliferation of cells in a concentration- and time-dependent manner, but the studied cell lines exhibited different susceptibilities. Table 1 summarizes the GR_50_ and IC_50_ values calculated in these cell lines for BSHE at different treatment times under assessment of cell vitality with TBDET and MTS assays. As expected, GR_50_ values were less dependent on BSHE exposure times than IC_50_ ones. For example, in HS27 normal fibroblasts, the GR_50_ value (MTS assay) was 106 μg/mL at 24 h and 71 μg/mL at 72 h of exposure, being the corresponding IC_50_ values 260 and 86 μg/mL, respectively. The values of GR_50_ and IC_50_ obtained for the studied cell lines differed depending on the above vitality assays. However, the three tumor lines were more responsive, whatever the vitality assay, than the normal HS27 line as shown by values of GR_50_ and IC_50_ for all times of exposure to BSHE to which, among tumor cells, BMel cells were the most responsive. In particular, the GR_50_ values at 48 h-exposure to BSHE (MTS assay) were 72, 48, 38, and 32 μg/mL for HS27, HCT 116, PC3 and BMel cells, respectively, while the corresponding IC_50_ values were 111, 46, 73, and 46 μg/mL (Table 1). Subsequent experiments were carried out by exposing the same cells for 24 and 48 h to these GR_50_ concentrations. At such concentrations, cells were 95% vital, being BSHE active mainly by inhibiting cell proliferation. It is to be pointed out that a 48 h-exposure to 125 μg/mL of BSHE caused detachment of all BMel and HCT 116 cells, which could be found only as fragments in the supernatant. On the other hand, HS27 and PC3 cells, when treated in the same way, remained viable in percentages of 47% and 33%, respectively, in comparison with the corresponding untreated cells, in the absence of quantizable cell fragments in the supernatant.

### 3.2 BSHE-induced autophagosome/autolysosome accumulation

**FIGURE 2:**
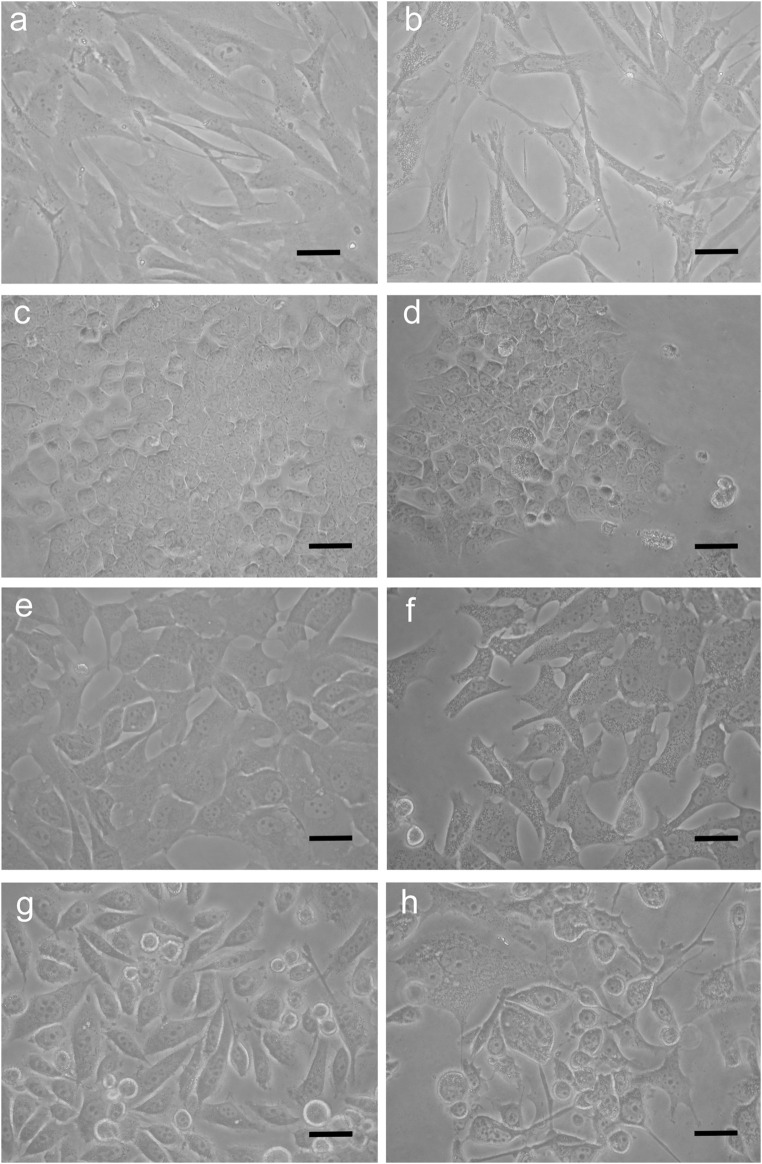
Representative inverted phase-contrast microscopy pictures of *Buxus sempervirens* hydroalcoholic extract (BSHE)-untreated cells **(A,C,E** and **G)** and cells treated **(B,D,F** and **H)** for 48 h with BSHE at GR_50_ concentrations of 72, 48, 38, and 32 μg/mL for HS27, HCT 116, PC3 and BMel cells, respectively). a,b: HS27 normal human skin fibroblasts; c,d: HCT 116 colorectal carcinoma cells; e,f: BMel melanoma cells; g,h: PC3 prostate cancer cells. BSHE-treated cells show vesicle accumulation in the cytoplasm mainly around nuclei. Scale bar, 40 μm.

**FIGURE 3:**
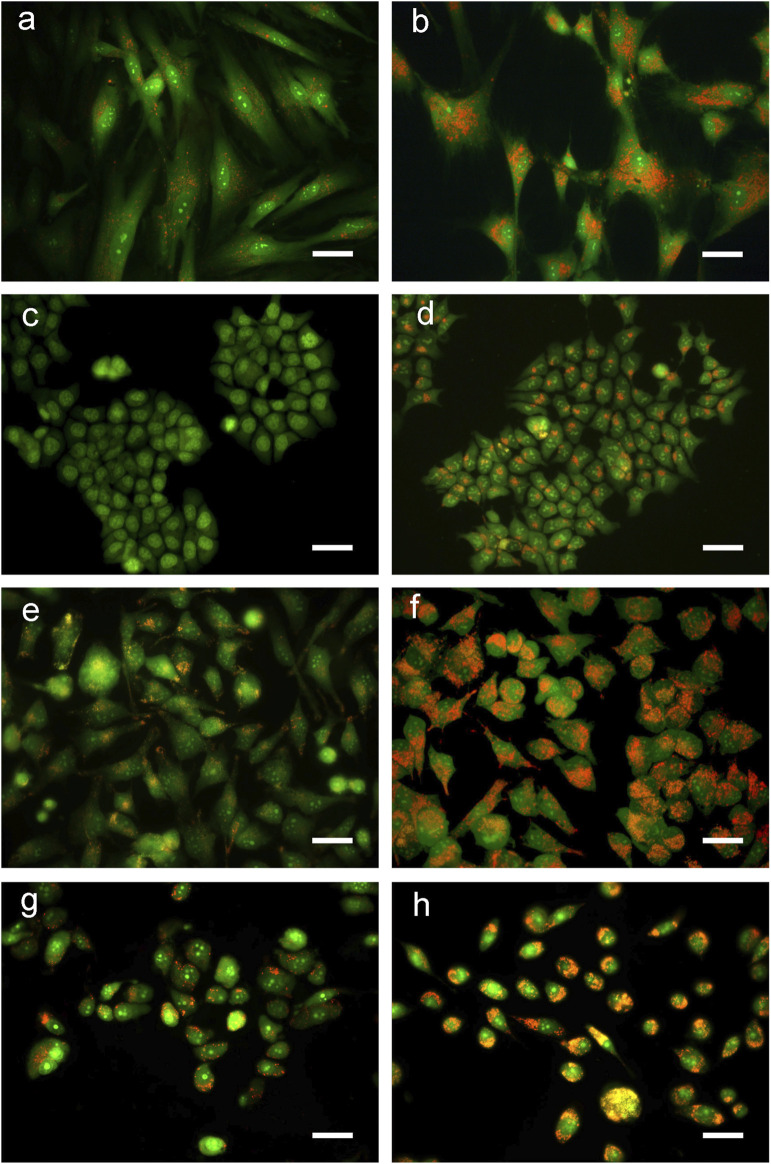
Representative fluorescence microscopy pictures of *Buxus sempervirens* hydroalcoholic extract (BSHE)-untreated cells **(A,C,E** and **G)** and cells treated **(B,D,F** and **H)** for 48 h with BSHE at GR_50_ concentrations of 72, 48, 38 and 32 μg/mL for HS27, HCT 116, PC3 and BMel cells, respectively. Cells were stained with acridine orange (AO) and images were acquired using a color camera. In live cells, AO is accumulated by acidic vesicles yielding strong orange or red signals. BSHE-treated cells show a great increase of these vesicles. Scale bar, 40 μm.

**FIGURE 4:**
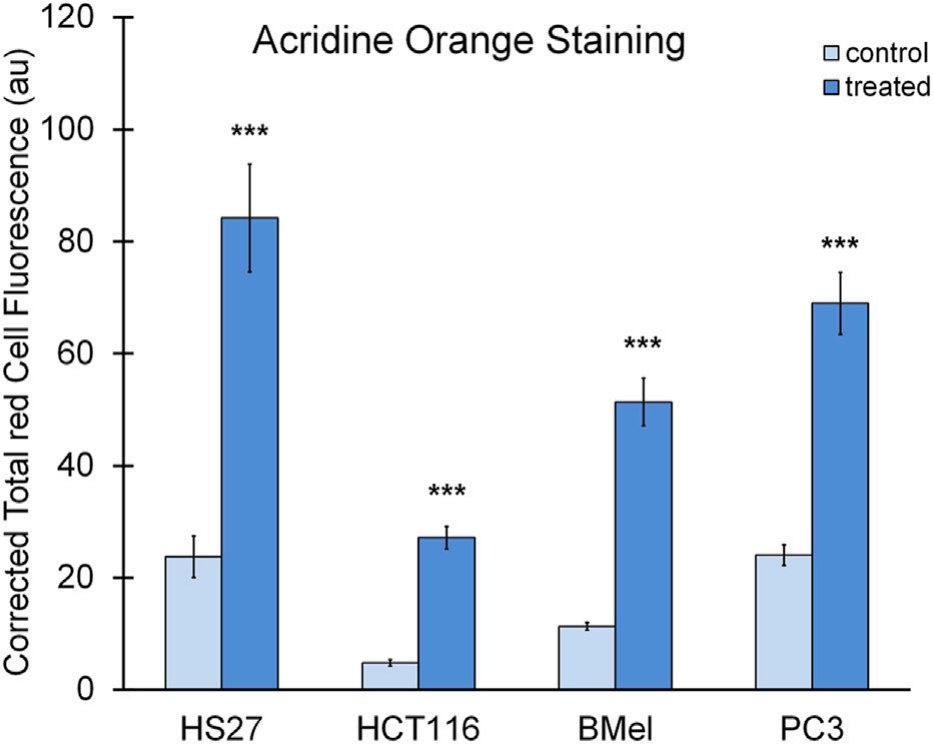
Quantification of red fluorescence in the BSHE-untreated (control) and -treated cells showed in Figure 3. Histograms represent corrected total red cell fluorescence (CTCF) as arbitrary units (au). CTCF, Integrated density—(Area of selected cell x mean fluorescence of background readings), see Materials and Methods. Cell fluorescence was measured by using the Fiji-ImageJ image processing software. Data presented are means ± S.E. (n = 50). ***p < 0.001 vs. control.

Cells, exposed for 48 h to BSHE (GR_50_) and observed under inverted phase-contrast microscopy, showed vesicle accumulation in the cytoplasm, mainly around nuclei (Figure 2). Following AO staining, these vesicles emitted red fluorescence revealing their acidic nature (Figure 3). In this respect, AO is known to freely enter cells, in which it is trapped and accumulated in acidic vesicles where, in a lower pH environment, its molecules are protonated, thus becoming unable to cross the vesicle membrane. At high concentrations, AO molecules form stacks and emit red fluorescence, whereas nucleic acid-bound AO characteristically emits green fluorescence (Pierzynska-Mach et al., 2014). In our experimental conditions, the red fluorescence in BSHE-treated cells increased significantly as compared to controls. In particular, such fluorescence was 5.4, 4.5, 3.5, and 2.9 higher in HCT, BMel, HS27 and PC3 cells, respectively (Figure 4).

**FIGURE 5:**
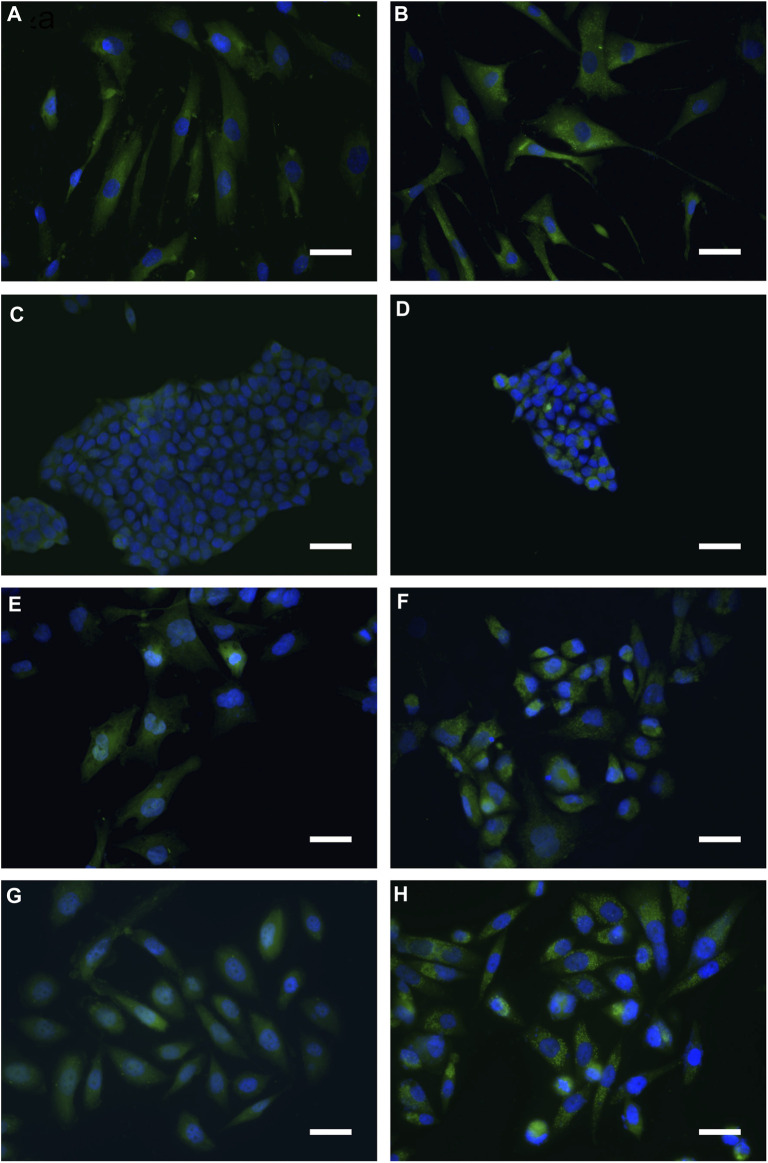
Representative indirect immunofluorescence images indicating accumulation of microtubule-associated light chain-3 protein (LC3) in *Buxus sempervirens* hydroalcoholic extract (BSHE)-untreated cells **(A,C,E** and **G)** and cells treated **(B,D,F** and **H)** for 48 h with BSHE at GR_50_ concentrations of 72, 48, 38, and 32 μg/mL for HS27, HCT 116, PC3 and BMel cells, respectively. Immunofluorescence analysis, carried out by using anti-LC3 antibodies, evidenced cell LC3 puncta accumulation following BSHE treatment. Nuclei are in blue (DAPI). Scale bar, 40 μm.

In order to assess whether the above vesicles were autophagosomes, we considered the microtubule-associated light chain-3 protein (LC3) that represents the most reliable marker protein for autophagy. LC3 is present in two forms: LC3I, distributed through the cytoplasm, and LC3II, phosphatidylethanolamine conjugate that is recruited in autophagosome membranes (Zhang et al., 2016). Immunofluorescent localization of LC3 in control cells showed that it was homogeneously spread within the cytoplasm, thus indicating the presence of the unprocessed form (LC3I). On the other hand, in all cells exposed to BSHE (GR_50_) for 48 h there were many dots close to nuclei corresponding to the processed, autophagosome-specific isoform of LC3 (LC3II) (Figure 5).

### 3.3 Autophagic flux markers evaluation in cells exposed to BSHE

LC3, as above reported, is the most widely used autophagosome marker to evaluate the autophagic flux. During autophagy, LC3I, the cytoplasmic form, is conjugated with phosphatidylethanolamine to form LC3II that is recruited in autophagosome membranes. Both LC3II/LC3I ratio and LC3II level increase with autophagosome formation (Klionsky et al., 2016).

**FIGURE 6:**
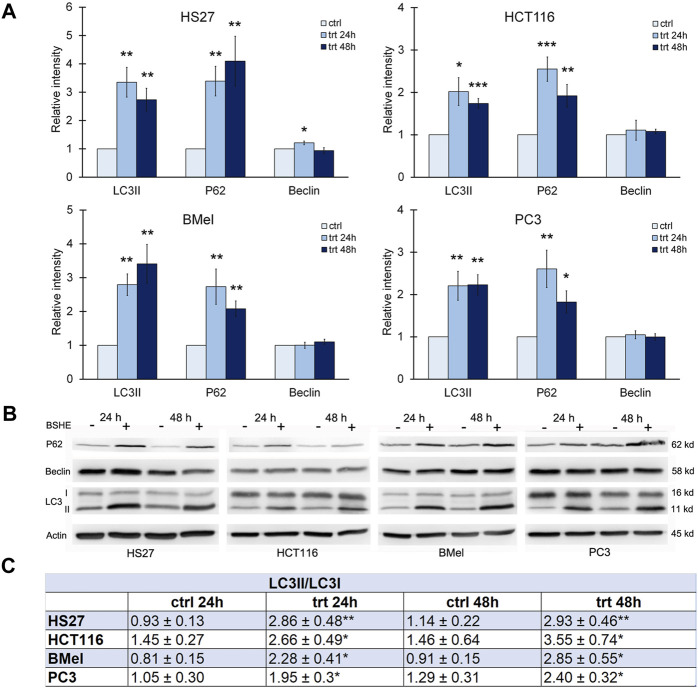
Effect of *Buxus sempervirens* hydroalcoholic extract (BSHE) on autophagic proteins levels in HS27, HCT116, PC3 and BMel cell lines following their treatment for 24/48 h with the respective GR_50_ concentrations (at 48 h) of BSHE (see Table 1). **(A)**: Histograms showing the relative intensity of the western blot bands of LC3II, p62 and beclin in BSHE-untreated (control, ctrl) and–treated cells (trt). Densitometry values were normalized for β-actin and compared with control. **(B)**: Representative western blots including the LC3-I ones. **(C)**: Table showing the LC3II/LC3I ratio, as sensitive index of autophagy, in the above BSHE-untreated and-treated cells; abbreviations: ctrl and trt as in **(A)**. Means ± S.E.M. are reported in A and C (n = 3 or more) where the Student’s t-test was employed to compare the values of BSHE-untreated and -treated cells. *p < 0.05; **p < 0.01; ***p < 0.001.

Densitometric analyses of western blots revealed that, when treated with BSHE (GR_50_) for 24 or 48 h, all cell lines showed statistically significant increases of LC3II protein level and LC3II/LC3I ratio, both representing a sensitive index of autophagy (Figure 6). There were no significant differences between the different cell lines nor between 24 and 48 h of BSHE exposure.

Sequestosome-1 or p62, an autophagic cargo protein, is another marker of autophagic flux. Autophagy induction involves, besides the increase of LC3II, a concurrent decrease of p62, which undergoes degradation during the autophagic process (Lao et al., 2014). In all cell lines treated for 24 or 48 h with BSHE, immunoblot analysis showed a statistically significant increase of p62 suggesting an inhibition of its autolysosome degradation with resulting autophagy unbalance (Klionsky et al., 2016) (Figure 6).

Also Beclin-1 is an important regulator of autophagy (Levine and Klionsky, 2004) being essential for formation and extension of the pre-autophagosomal structure. Immunoblot analysis showed that, in HS27 cells, Beclin-1 was upregulated following 24 h-treatment with BSHE, whereas its expression at 48 h did not differ from that of control. This finding is likely to be consistent with a role of Beclin-1 in early events of autophagy. However, in all other cell lines, no changes in the expression levels of Beclin-1 by BSHE were observed according to studies dealing with Beclin-1-independent mechanisms of autophagy in cancer cells (Smith et al., 2010; Athamneh et al., 2017; Pan et al., 2019) (Figure 6).

### 3.4 Effect of BSHE on cell cycle regulators and apoptotic proteins

**FIGURE 7:**
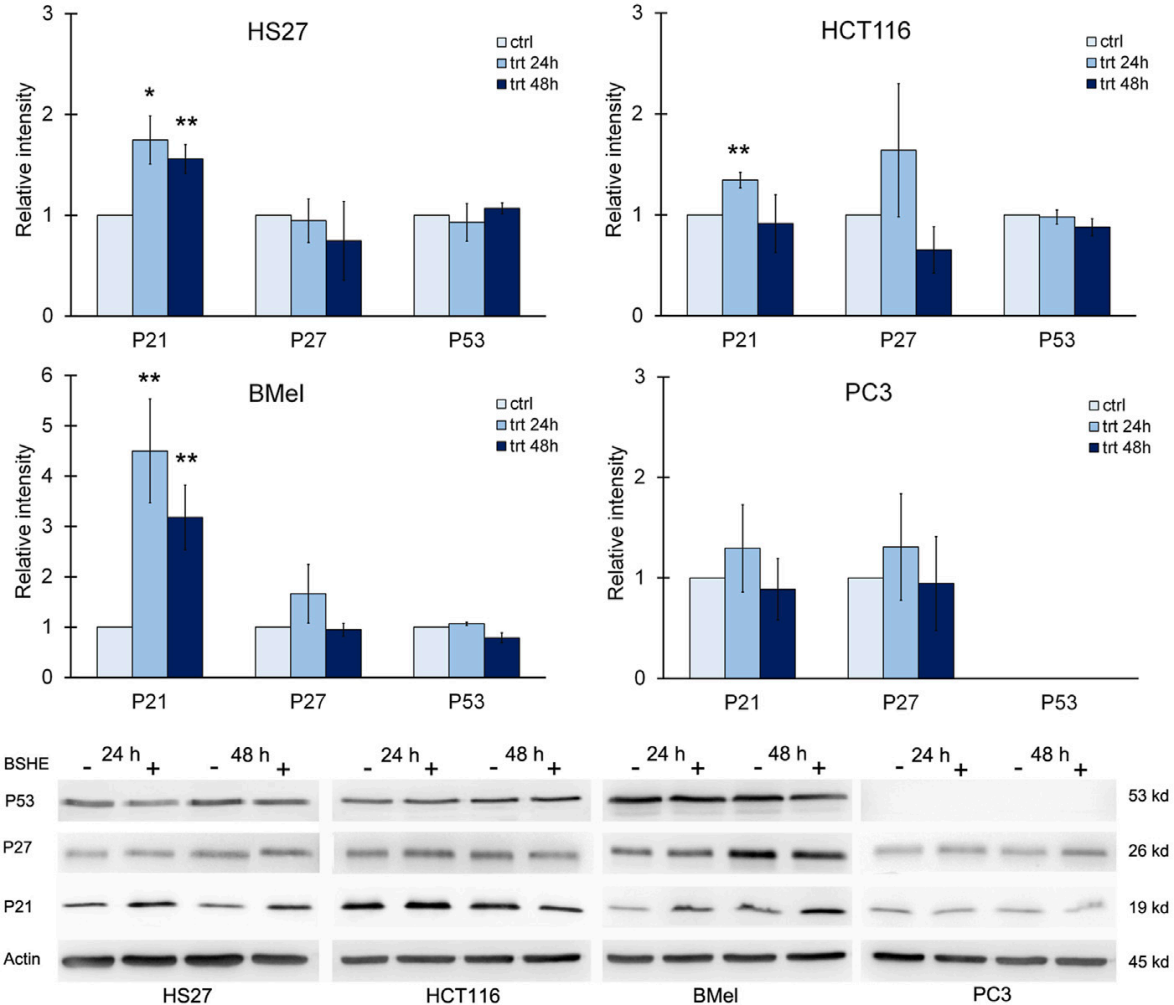
Effect of *Buxus sempervirens* hydroalcoholic extract (BSHE) on p21, p27 and p53 levels in HS27, HCT116, PC3 and BMel cell lines following their treatment for 24/48 h with the respective GR_50_ concentrations (at 48 h) of BSHE (see Table 1). Histograms showing relative intensity of the western blot bands of these proteins in BSHE-untreated (control, ctrl) and-treated cells (trt) along with representative western blots are shown. Densitometry values were normalized for β-actin and compared with control. Means ± S.E.M. are reported (n = 3 or more). The Student’s t-test was employed to compare the values of BSHE-untreated and -treated cells. *p < 0.05; **p < 0.01.

Progression through the cell cycle is regulated by cyclins and cyclin-dependent kinases (CDKs), being p21 (Wafl/Cip1) a CDK inhibitor that can induce G1 arrest. p21, known as a tumor suppressor, is a key mediator of p53-dependent cell cycle arrest after DNA damage that is transcriptionally controlled by p53 and p53-independent pathways (Gartel et al., 1996; Kreis et al., 2019). In the present study, immunoblot analysis showed, in comparison with the untreated cells, a statistically significant increase of p21 in HS27 fibroblasts and BMel melanoma cells exposed for 24 h or 48 h to BSHE. In HCT 116 cells, p21 increased following 24 h of treatment with BSHE without changes after 48 h. Moreover, no changes of p21 levels were observed in BSHE-exposed PC3 cells (Figure 7).

With regard to p53, it was expressed in all cell lines with the exception of PC3 ones according to the reported deletion of such protein in this cell line (Nguyen et al., 2005). However, treatment with BSHE did not modify p53 expression in the studied cells (Figure 7).

As shown in Figure 7, p27 (Kip l), another CDK inhibitor, did not vary significantly in all studied cell lines after treatment with BSHE.

**FIGURE 8:**
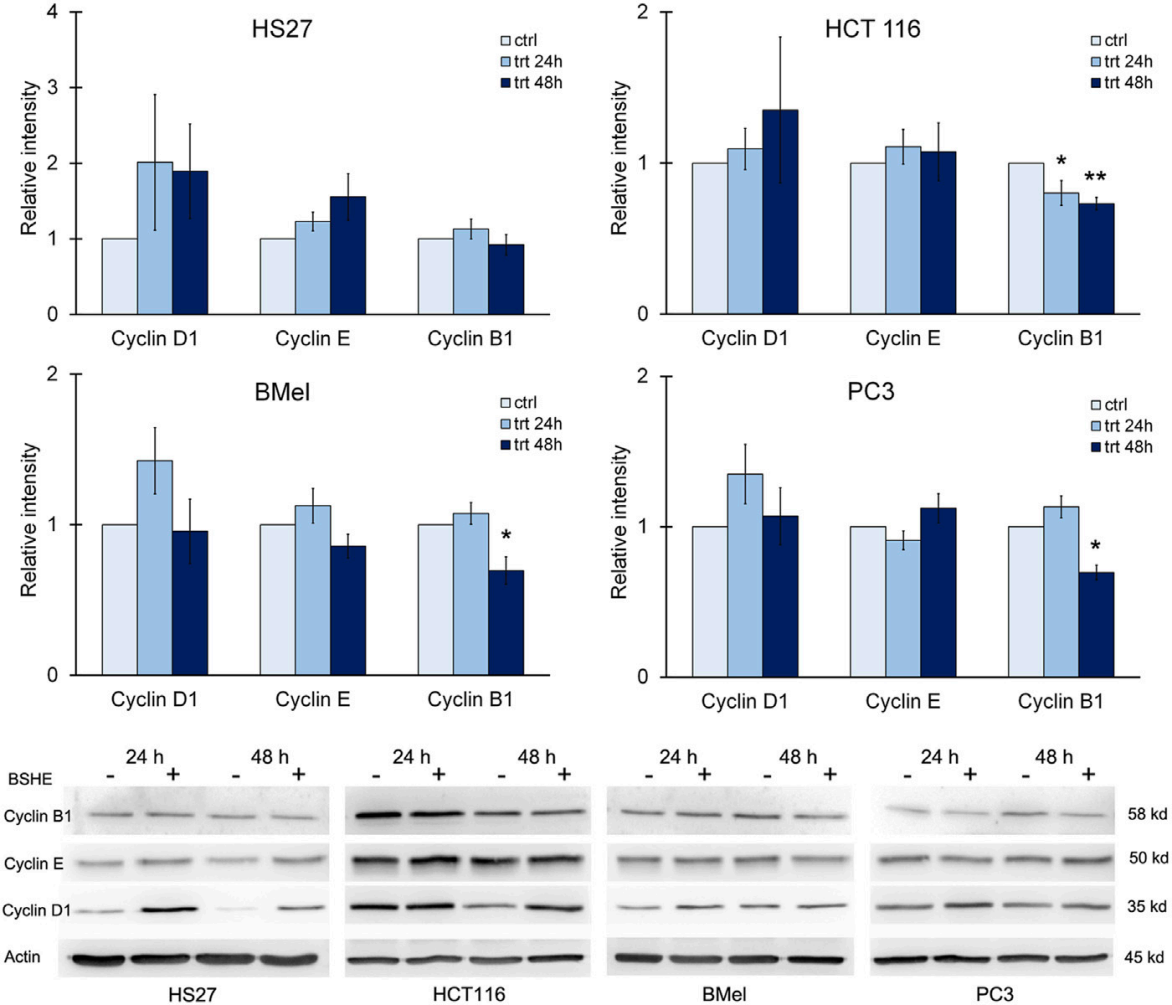
Effect of *Buxus sempervirens* hydroalcoholic extract (BSHE) on cyclins D1, E and B1 in HS27, HCT116, PC3 and BMel cell lines following their treatment for 24/48 h with the respective GR_50_ concentrations (at 48 h) of BSHE (see Table 1). Histograms showing relative intensity of the western blot bands of these proteins in BSHE-untreated (control, ctrl) and–treated cells (trt) along with representative western blots are shown. Densitometry values were normalized for β-actin and compared with control. Means ± S.E.M. are reported (n = 3 or more). The Student’s t-test was employed to compare the values of BSHE-untreated and -treated cells. *p < 0.05; **p < 0.01.

When considering cyclins, we evaluated the effects of BSHE treatment on levels of cyclin D1, cyclin E and cyclin B1. Immunoblot analysis revealed that cyclin B1 decreased significantly in HCT116 cells after 24 h and 48 h of treatment and only after 48 h in BMel and PC3 cells. These data agreed with the BSHE-induced inhibition of proliferation. On the other hand, an unexpected but not significant increase of cyclin D1 by BSHE was found in all tested cells, while the expression of cyclin E did not vary significantly in all BSHE-treated cells (Figure 8).

**FIGURE 9:**
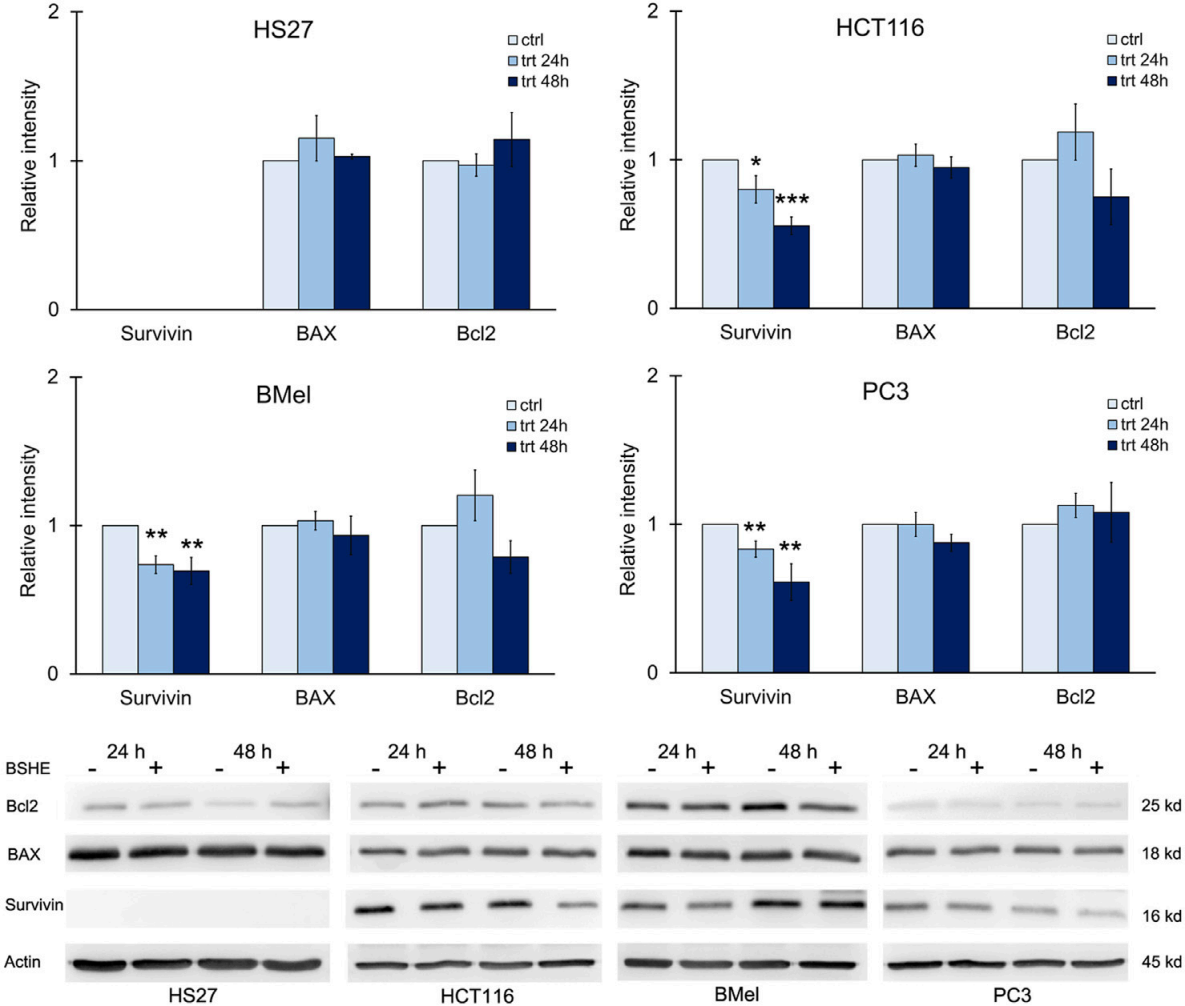
Effect of *Buxus sempervirens* hydroalcoholic extract (BSHE) on levels of the apoptotic proteins survivin, BAX and Bcl2 in HS27, HCT116, PC3 and BMel cell lines following their treatment for 24/48 h with the respective GR_50_ concentrations (at 48 h) of BSHE (see Table 1). Histograms showing relative intensity of the western blot bands of these proteins in BSHE-untreated (control, ctrl) and -treated cells (trt) along with representative western blots are shown. Densitometry values were normalized for β-actin and compared with control. Means ± S.E.M. are reported (n = 3 or more). The Student’s t-test was employed to compare the values of BSHE-untreated and -treated cells. *p < 0.05; **p < 0.01; ***p < 0.001.

We also evaluated the expression of survivin, an anti-apoptotic protein. It is highly expressed in most human cancers and during fetal development, while being present at low levels or completely absent in healthy cells and tissues. Its expression levels correlate with more aggressive disease and poor clinical outcome. Within cell cycle, survivin is highly expressed at G2/M phase and declines rapidly in G1 phase (Jaiswal et al., 2015). Immunoblot analysis revealed that HCT116, BMel and PC3 cell lines, when treated with BSHE (GR_50_) for 24 h or 48 h, showed a significant decrease in survivin expression, whereas HS27 human fibroblasts did not express such protein. BAX and Bcl_2_ (proapoptotic and antiapoptotic proteins, respectively) did not vary following BSHE treatment of the studied cells (Figure 9).

## 4 Discussion

Autophagy is a cellular process by which intracellular proteins and organelles are sequestered and degraded after fusion of autophagosomes with lysosomes. The process starts with formation of the phagophore, which originates from endoplasmic reticulum, Golgi apparatus or plasma membrane (Mari et al., 2011). Phagophore engulfs a portion of cytoplasm, thereby sequestering proteins and organelles in a double membrane structure named autophagosome (Mijaljica et al., 2012). The outer membrane of autophagosome fuses with lysosomal membrane to form an autolysosome, while the autophagosomal inner membrane is degraded by lysosomal acid proteases together with the sequestered materials. Finally, the degradation products are transported to the cytoplasm to be recycled (Zhang et al., 2016), being the whole process called “autophagic flow”.

A set of autophagy-related proteins, including the microtubule-associated light chain-3 protein (LC3), plays a critical role in regulating autophagosome formation. Upon autophagy induction, the cytosolic LC3 (LC3I) is conjugated to phosphatidylethanolamine to form lipophilic LC3 (LC3II) that binds to the expanding autophagosome membrane while remaining bound to the completed autophagosome. Therefore, LC3II is taken on a marker of autophagosome (Mizushima et al., 2010).

In addition to LC3, levels of p62 are also used to monitor autophagic flux. p62 possesses a C-terminal ubiquitin-binding domain and a short LC3-interacting region sequence (Lippai and Low, 2014.), thus serving as an adaptor protein that links ubiquitinated proteins to the autophagic machinery and enables their clearance in lysosomes (Bjorkoy et al., 2009). p62 and p62-bound ubiquitinated proteins become incorporated into the completed autophagosome and are degraded in autolysosomes. p62 is autophagy substrate, being degraded along with the cargo proteins in normal cells and accumulated under autophagy deficiency, thus serving as potential biomarker for defective autophagy.

Arrest of proliferation and death were observed in all cell lines exposed to BSHE with, however, different susceptibilities to the extract as shown by its GR_50_ and IC_50_ values. Melanoma cells were the most susceptible where, following 48 h of exposure, GR_50_ and IC_50_ (MTS assay) for BSHE were, respectively, 32 and 46 μg/mL. On the other hand, normal fibroblasts were the cell line less susceptible to BSHE whose GR_50_ and IC_50_ (MTS assay) at 48 h of exposure were, respectively, 72 and 111 μg/mL, whereas colorectal carcinoma cells and prostate cancer cells had an intermediate sensitivity. The studied cells exposed for 48 h to BSHE (GR_50_) remained almost all vital but higher concentrations of this extract or longer times of exposure to it caused cell death.

Immunoblot analysis of cell cycle proteins showed that, following 24/48 h treatment with BSHE (GR_50_), p21, a CDK inhibitor transcriptionally controlled by p53 that can induce G1 arrest, increased in all cell lines except for PC3 prostate cancer cells. Such result agrees with the previously reported p53 deletion in PC3 cells (Nguyen et al., 2005). Moreover, with regard to cyclins, only a decrease in cyclin B1 was found in all cell lines except for HS27 normal fibroblasts. Since cyclin B1 is known to increase just before mitosis, its above decrease agrees with the BSHE-induced inhibition of cell proliferation. As for proteins involved in apoptosis, BSHE did not change BAX and BCl_2_ levels in the studied cells while decreasing, however, that of the antiapoptotic protein survivin already after 24 h of treatment in BMel, HCT116 and PC3 cells. HS27 cells, as expected from healthy cells (Jaiswal et al., 2015), appeared to not express survivin. Therefore, both increase in p21 and decrease in survivin suggest that the BSHE-treated cells underwent G1 arrest.

A well evident effect was the accumulation of vesicles in the cytoplasm of BSHE-treated cells. This effect was documented after 48 h of treatment, being anyhow clearly present after 24 h. Vesicles had an acidic nature, as evidenced by their red color following AO staining, while immunofluorescence analysis using anti-LC3 antibodies revealed, in these vesicles, a characteristic LC3II pattern as typically observed after LC3 (LC3I) recruitment by autophagosome membranes. Such pattern was not present in the studied BSHE-untreated cells, which only showed a diffuse fluorescence. Therefore, the above vesicles could be thought to be autolysosomes. These results, along with the increase of both LC3II and LC3II/LC3I ratio evidenced by Western immunoblot analysis, appeared to suggest a BSHE-induced activation of autophagy. On the other hand, a marked increase of p62 was observed in all cell lines after 24–48 h of treatment with BSHE (GR_50_). p62, incorporated in autophagosomes, is known to decrease in a normal autophagic process when autophagy flux increases owing to its degradation in autolysosomes together with the other cargo proteins. Therefore, the observed p62 accumulation indicated a BSHE-dependent block in these autophagosome degradation processes.

Autophagy is a dynamic process including LC3 accumulation and LC3II formation indicating an increased autophagosome generation (i.e., autophagy induction) or, alternatively, a block in the downstream phases of autophagy (Rubinsztein et al., 2009; Zhang et al., 2016). If, in the BSHE-treated cells, the increased levels of p62 were likely to reveal a block in the evolving autophagic process, the BSHE-induced LC3II increase was likely to suggest a concomitant effect of autophagy induction. In this regard, BSHE-exposed HS27 fibroblasts showed, in addition to the increase in LC3II, upregulation of Beclin 1, an autophagic protein essential for both formation and extension of the pre-autophagosomal structures.

A vast literature has dealt with the complex relationships between autophagy and cancer. Depending on cell type and stage, autophagy may play a dual role in cancer by acting as tumor suppressor or promoter of tumor progression. In early stages, autophagy usually acts as tumor suppressor allowing the cells to discard the damaged components whereas, in advanced stages, it acts as tumor promoter by favouring cell survival in conditions of low availability of oxygen and nutrients (Gracio et al., 2017). In many cases, cancer cells are more dependent on autophagy than normal cells, probably due to increased metabolic demands (White, 2015), being autophagy essential for cell survival mostly in hypoxic tumor regions with stimulus to tumor growth, invasion and metastatic diffusion (Guo et al., 2013). Autophagy may play a dual role also in the cancer response to many antineoplastic drugs (Thorburn et al., 2014). Among them, some drugs increase autophagic flow or induce accumulation of autophagosomes without affecting autophagic flow, while other agents can both increase the formation of autophagosomes and block their fusion with lysosomes (Köchl et al., 2006; Ganley et al., 2011; Thorburn et al., 2014). In many cases, autophagy activation is necessary for the antitumor effect and, consequently, inhibition of autophagy by drugs reduces tumor cell death with increased tumor growth. For example, Erlotinib [an EGFR inhibitor used in the first line treatment of not small cell metastatic lung cancer (NSCL) showing exon deletions or mutations of EGFR] requires autophagy activation to maximize inhibition of tumor growth (Wei et al., 2013). Conversely, extensive literature suggests that autophagy can protect cancer cells from antineoplastic therapy (Thorburn et al., 2014; Towers and Thorburn, 2016). For example, in melanoma resistance to Vemurafenib, a BRAF kinase inhibitor, there is an increase in autophagy whose inhibition can reverse resistance to this drug (Ma et al., 2014).

## 5 Conclusion

The present study shows that BSHE induced arrest of cell proliferation and death with accumulation of autophagosomes/autolysosomes and unbalance of autophagic flux. Such effects were observed in all BSHE-treated cells but BMel melanoma ones were those responsive to lower concentrations of the extract (whose GR_50_ value was about half of that of HS27 normal fibroblasts). The obtained evidences give credit to BSHE as a potential and valuable source of new drugs able to act against melanoma and other aggressive and treatment-resistant cancers and also to counteract the autophagy-dependent resistance to particular antineoplastic compounds. Apart from cyclovirobuxine D, we are unaware of studies dealing systematically and specifically with the wide ensemble of compounds present in *B. sempervirens*. On the other hand, further studies are needed to clarify the molecular mechanisms by which bioactive compounds from BS can induce autophagy with, however, subsequent inhibition of autophagosome degradation.

## Data Availability

The raw data supporting the conclusions of this article will be made available by the authors, without undue reservation.
